# High saturated fat and low carbohydrate diet decreases lifespan independent of body weight in mice

**DOI:** 10.1186/2046-2395-2-10

**Published:** 2013-06-03

**Authors:** Alexandre Pastoris Muller, Marcelo de Oliveira Dietrich, Adriano Martimbianco de Assis, Diogo Onofre Souza, Luis Valmor Portela

**Affiliations:** 1 Departamento de Bioquímica, ICBS, Universidade Federal do Rio Grande do Sul, Ramiro Barcelos, 2600 – Anexo I, Porto Alegre, RS, 90035-000, Brazil; 2 Programa de Pós-graduaçãoemBioquímica, ICBS, Universidade Federal do Rio Grande do Sul, Ramiro Barcelos, 2600 – Anexo I, Porto Alegre, RS, 90035-000, Brazil; 3 Program in Integrative Cell Signaling and Neurobiology of Metabolism, Section of Comparative Medicine, Yale University School of Medicine, New Haven, CT, 06520, USA

**Keywords:** Obesity, Glucose tolerance, High-fat diet, Memory, Survival

## Abstract

**Background:**

Obesity is a health problem that is reaching epidemic proportions worldwide. We investigated the effects of a life-long high saturated fat and low carbohydrate (HF) diet on the body mass, glucose tolerance, cognitive performance and lifespan of mice.

**Findings:**

C57BL/6J mice were fed with a HF diet (60% kcal/fat) or control diets (15% kcal/fat) for 27 months. One-half of the mice on the HF diet developed obesity (diet-induced obese (DIO) mice), whereas the remaining mice were diet resistant (DR). At 8 months of age, both DIO and DR groups had increased hyperglycemic response during a glucose tolerance test, which was normalized in 16-month-old mice. At this latter time point, all groups presented similar performance in cognitive tests (Morris water maze and inhibitory avoidance). The survival curves of the HF and control diet groups started to diverge at 15 months of age and, after 27 months, the survival rate of mice in the DIO and DR groups was 40%, whereas in the control diet group it was 75%.

**Conclusions:**

AHFdiet decreased the survival of mice independent of bodyweight.

## Findings

### High-fat diet decreases survival

Obesity and overweight are associated with numerous co-morbidities, including type 2 diabetes and neuropsychiatric disorders [1-3]. A sedentary lifestyle and excessive consumption of diets enriched in saturated fat and/or high glucose contribute to the onset and progression of these co-morbidities [4,5] leading to decreased life expectancy [6,7]. The effects of increased ingestion of dietary fat and carbohydrate are well stabilized in rodent models, leading to obesity and disturbed glucose metabolism, similar to that seen in humans [8-10].

The ingestion of high-fat diets, rich in saturated fat, lead to metabolic and neurochemical alterations, which contribute to impaired performance of rodents in a variety of behavioral tasks [8,11]. Both peripheral and central perturbations, including hyperglycemia, altered expression of synaptic proteins and signaling proteins, are among the changes implicated in the deterioration of the cognitive function [6].

One rodent strain that is particularly susceptible to the effects of dietary fat is the C57BL/6 mouse [12]. C57BL/6 mice develop severe obesity, hyperglycemia and hyperinsulinemia when fed a high-fat diet. However, when fat content is within the normal range animals remain lean and euglycemic [13]. Interestingly, about 50% of C57BL/6 mice do not become obese when fed with high-calorie diets [12,13] indicating individual differences in terms of bodyweight gain under such dietary conditions despite similar environment and congenic genetic background [14,15].

In the present study, we aimed to identify changes associated with consumption of alife-long high saturated fat and low carbohydrate (HF) diet on glucose tolerance, memory performance and life expectancy in C57BL/6 mice, comparing those mice that developed obesity (diet-induced obesity, DIO) to those that were resistant to obesity (diet resistant; DR).

## Availability of supporting data

### Animals and diet

Twenty-five C57Bl6 male mice, aged 21 days, were randomly placed on one of two different diets for 27 months: a control diet (CD, n = 12) or aHFdiet (n = 13). The HF diet contained 60% energy from saturated and unsaturated fat (45% lard and 15% soybean oil), 15% energy from starch (corn) and 25% from protein (soybean protein). CD contained 15% energy from saturated fat and unsaturated fat (soybean oil), 60% energy from starch (corn) and 25% from protein (soybean protein). Both diets were formulated in our laboratory and contained standard vitamins and minerals mixed with all essentials nutrients. Diets in the form of pellets and water were provided *ad libitum*[7]. Animals were weighedevery week during treatment. Mice were housed in standard cages (20 × 15 cm), four animals per cage, in a room with a controlled temperature (22°C) under a 12-hour light/12-hour dark cycle. All experiments were in agreement with the Committee on Care and Use of Experimental Animal Resources, Universidade Federal do Rio Grande do Sul, Brazil.

### Glucose tolerance test

At 8 and 16 months of age, mice were fasted for 12 hours and then received an intraperitoneal (i.p.) injection of glucose (2 mg/g body weight). Blood was drawn from the tail and glucose levels was measured by a glucosimeter at time 0 (fasting) and at 30, 60 and 120 minutes post-glucose injection (AccuChek Active, Roche Diagnostics®, USA).

### Morris water maze task

At 15 months of age, mice were subjected to a spatial memory task as previously described [16]. The water maze apparatus was a black circular pool (110 cm diameter) with the water temperature maintained at 21 ± 1°C. Mice were trained with two trials per day for 5consecutive days, each trial lasting up to 60 seconds with 20 seconds of rest on a hidden black platform. During training, mice learned to escape from the water by finding a hidden rigid black platform submerged approximately 1 cm below the water surface in a fixed location and, if it failed to find the platform in 60 seconds, it was placed gently on the platform and allowed to rest for 20 seconds. The maze was located in a well-lit white room with several visual stimuli hanging on the walls to provide spatial cues. Decreased time latency to escape during each trial was measured as an indicator of learning. On the sixth day, a probe trial was performed without the platform and the time spent in the target quadrant was used as a measure of retention memory.

### Inhibitory avoidance task

At 15 months of age, mice were also submitted to an aversive memory task. The apparatus was a 50 × 25 × 25 cm acrylic box with a floor of parallel caliber stainless steel bars (1 mm diameter) spaced 1 cm apart (Insight Equipamentos, SP, Brazil). Mice were placed on the platform located in the center of the apparatus and their latencies to step down on the floor with all four paws were measured with an automated device. During the training sessions, when animals stepped down onto the grid, they received a 1-second, 0.4-mA foot shock and were immediately returned to their home cages. The test sessions were performed without foot shock at 2 and 24 hours after training to evaluate short- and long-term memory, respectively. The mice were returned to the platform and the latency to step down (180 seconds maximum) was used as a measure of retention [17]. A foot shock was omitted during testing sessions. Data from the inhibitory avoidance task are shown as median (interquartile ranges) of latencies to step down on the grid in both the test and training sessions.

### Statistical analysis

Results are expressed as means ± S.E.M. except for data from the inhibitory avoidance task, which is represented by median and interquartile range. The data from the water maze acquisitions task, glucose tolerance test (GTT) and bodyweight were analyzed using a repeated-measures analysis of variance (ANOVA), followed by Duncan’s *post***-***hoc* test. Data from the inhibitory avoidance task were analyzed by a Mann–Whitney *U* test. Differences between groups were considered statistically significant at *P*< 0.05. Cumulative survival probability was plotted on a Kaplan–Meier curve with pairwise comparisons of diets using the log-rank statistic analysis.

## Bodyweight

**Figure 1:**
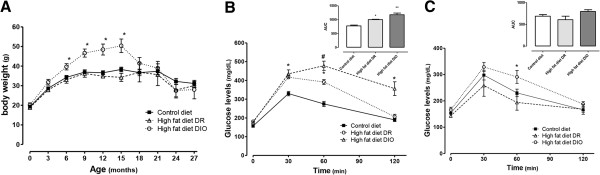
**Body weight curves and glucose tolerance test.** (**A**) Body weight curves (means ± S.E.M.) during the length of the study (*P< 0.05,DIO> DR and CD). The glucose tolerance test (GTT) was performed (**B**) at 8 months (*P< 0.05, DIO and DR > CD; #*P*< 0.05, DIO > DR and CD) and (**C**) 15 months after diet introduction (*P< 0.05, DIO > DR and CD; CD, n =12; high saturated fat and low carbohydratediet: DIO, n = 7; DR, n = 6). Blood glucose levels (means ± S.E.M.) were assessed at fasting (0 minutes), 30, 60 and 120 minutes after an intraperitoneal glucose injection. Insert: area under the curve (AUC) of the GTT.CD, control diet; DIO, diet-induced obese; DR, diet resistant.

As previously described by others [12,13], approximately 50% of the mice fed a HF diet became obese, whereas the other 50% had similar bodyweight compared to aged-matched control animals fed a control diet (Figure 1A). Based on this data set, we stratified animals fed a HF diet intoDR or DIO groups. Mice in the CD and DR groups increased bodyweight for up to 9 months, which was maintained up to 21 months of age. DIO mice still increased body weight for up to 15 months of age, and then this declined to reach the weight of the CD and DR groups at 18 months. The body weight of DIO mice was statistically different from other groups from 6 to 15 months (*P*< 0.05, Figure 1A).

### Glucose tolerance test

Next, we assessed glucose tolerance after 8 and 16 months of diet treatment. After 8 months of HF, both DIO and DR mice had increased fasting blood glucose levels (DR and DIO > CD, *P*< 0.05) and a robust hyperglycemic response during GTT relative to CD (DR and DIO > CD, *P*< 0.05). In addition, DIO mice had long-lasting hyperglycemia compared to CD and DR groups (120 minutes DIO > CD and DR, *P*< 0.05, Figure 1B). DR and DIO had increased area under the curve of GTT (DIO > DR > CD, *P*< 0.05; Figure 1B insert). After 16 months on diet, the glucose profile during GTT was attenuated: DIO mice showed an impaired GTT response only at 60 minutes post i.p. glucose administration (*P*< 0.05, Figure 1C), and the area under the curve was not statistically different among groups (Figure 1C, insert).

### Cognition tasks

**Figure 2:**
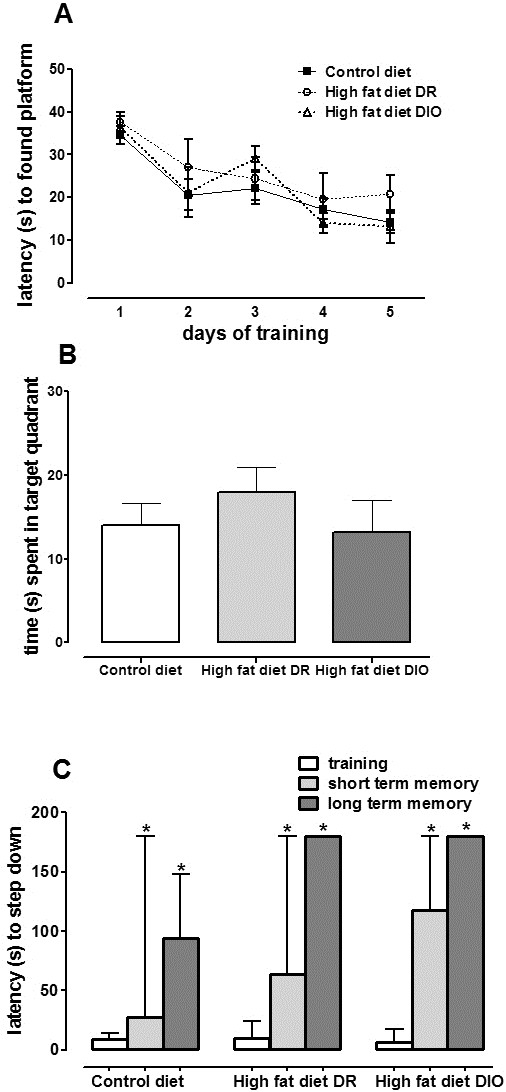
**Performance on memory tasks after 16 months of treatment.** (**A**) Latency to find the platform during the acquisition phase of the Morris water mazetest (means ± S.E.M.) and (**B**) time spent in the target quadrant during the probe task (means ± S.E.M.). (**C**) Inhibitory avoidance task latency to step down onto the platform (median/interquartile ranges) during training and at the short- and long-term memory tests. Controldiet(n = 12) and high fat diet groups (diet-induced obese (DIO), n = 7; diet resistant (DR), n = 6). *P< 0.05, test sections > training section.

Spatial (Morris water maze; MWM) and aversive (inhibitory avoidance) memory was evaluated in 14- to 15-month-old mice. In the MWM task, there was no significant effect of HF diet on latency to find the platform during the acquisition phase and probe trial (Figure 2A,B). In the inhibitory avoidance task, all groups showed an increased latency to step down the platform in the training and test sessions (*P*< 0.05, Figure 2C) with no differences among groups.

### Survival

**Figure 3:**
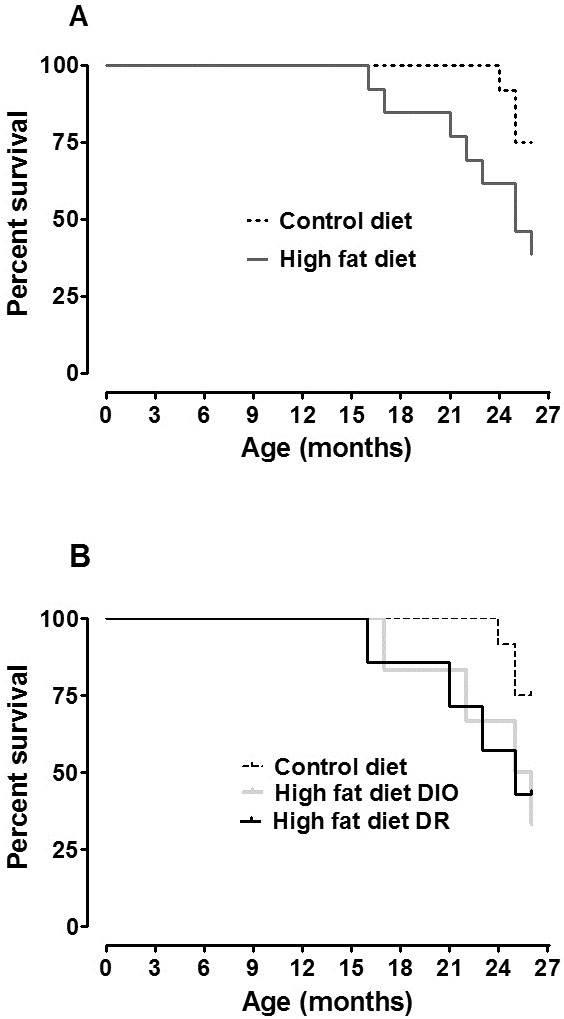
**Kaplan–Meier survival curves over 27 months.** (**A**) Survival based on treatment. Control diet (initial n = 12; final n=9) and high fat diet (initial n = 13; final n = 5). (**B**) Survival based on phenotype (initial diet-induced obese (DIO), n = 7; final n=2; initial diet resistant (DR), n = 6; final n=3).

At 15 months of age, the survival curves of the HF groups and the CD group started to diverge and remained separate until the end of treatment (Figure 3A). At 27 months of age, the treatment was stopped. The survival rate of HF mice (DIO and DR) was 40% and that of the control diet mice was 75%. There was no statistical difference in the survival rate between the DIO and DR mice (Figure 3B).

The present study demonstrate that mice fed a HF diet develop different phenotypes with regards to body weight (DIO and DR) and glucose tolerance (impaired at 8 months and almost normal at 16 months), but showed similar performance in memory tasks (spatial and aversive) and mortality rate. Moreover, the increasing mortality rate was dependent on dietary saturated fat composition but independent of body weight gain.

As previously shown by others [14,15], we observed that C57BL/6 mice fed a HF diet were either DR or DIO related to body weight gain. The body weight gain observed in DIO mice increased until mice were 15 months old, similarly to Baur *et al*. [18]. However, the normal age-associated body weight decline was greater in our study and preceded those reported by Baur *et al*. [18].

Obesity is a key factor involved in the pathology of diabetes and a risk factor to cardiovascular and cerebrovascular diseases. Mice fed with HF diets up to 8 months were more resistant to a glucose load compared to CD-treated mice. Blood glucose levels were elevated in DIO mice during the whole course of the GTT, whereas DR mice had increased glucose levels at fasting, 30 and 60 minutes during the GTT compared to CD mice, despite their lean phenotype. At 16 months age, animals appear to recover their ability to regulate peripheral glucose levels; however, at this time-point we cannot rule out an effect of age in glucose metabolism [19]. Moreover, subtle alterations can take place in the early stages of high saturated fat intake and can progress throughout the course of years, modifying insulin levels and carbohydrate metabolism or insulin receptor response, thus affecting peripheral tissues and brain metabolism.

A variety of studies have assumed that excessive HF consumption causes obesity and contributes to disturbances

 in synaptic machinery, with deleterious consequences for neuronal signaling, neuron-to-glia connectivity and cognitive functions [11,20]. Despite increased body weight at the time of behavioral tasks along with impaired glucose metabolism at earlier phases of HF, surprisingly DIO and DR mice showed no cognitive decline in spatial or aversive

 memory tasks compared to CD mice. Differences in time of regimen, diet composition, strain of animals and behavioral protocols may potentially account for these disparities. Moreover, considering that a HF diet-induced hyperglycemia during the early stages of treatment may cause synaptic alterations we cannot rule out memory deficits after 15 month of age

In addition to investigating the detrimental effects of a HF diet on cognitive function, we also examined its implication on lifespan. Although the association of obesity with increased risk of cardiovascular disease and diabetes is well known, it is often under-appreciated that the risks of other age-related diseases, such as cancer and inflammatory disorders, are also increased. Conversely, reducing caloric intake by 40% below that of *ad libitum*-fed animals (caloric restriction) is the most robust and reproducible way to delay age-related diseases and extend lifespan in mammals [21]. In this work, we demonstrated that mice fed with a HF diet for 27 months, *ad libitum*, had a decreased life expectancy and further, when this group was stratified into DIO and DR groups, the life expectancy curve was similar. Similarly, Baur *et al*. [18] reported that a high calorie diet (60% from fat) caused a deleterious impact on general health status and survival rate in middle-aged mice. We were not able to explain the exact mechanism by which a HF diet increased mortality; however, the high energy source from saturated fat over 27 months could be implicated in increased reactive oxygen species production and oxidative stress, which is the main process responsible for aging and also a contributory factor for neurodegeneration [22,23]. Also, activation of inflammatory and immunological response induced by a HF diet and/or aging may exert a negative impact and be implicated in the differences in life expectancy observed in our study [21,24,25].

In conclusion, C57BL/6 mice fed a HF diet may develop an obese phenotype or not compared to mice fed a CD. A HF diet decreased the survival rate independent of body weight gain.

## Abbreviations

ANOVA: Analysis of variance; CD: Control diet; DIO: Diet-induced obese; DR: Diet resistant; GTT: Glucose tolerance test; HF: High saturated fat and low carbohydrate; i.p: Intraperitoneal; MWM: Morris water maze

## Competing interests

The authors declare that they have no competing interests.

## Authors’ contributions

APM: Performed experiments, analyzed data and wrote the manuscript. MOD: Analyzed data and wrote the manuscript. AMA: Performed experiments and analyzed data. DOS: analyzed data and wrote the manuscript; LVP: analyzed data and wrote the manuscript. All authors read and approved the final manuscript.
